# Rosmarinic Acid Enhances Functional Recovery of Injured Muscle via Reducing Fibrosis and Enhancing Muscle Regeneration After Muscle Contusion

**DOI:** 10.7150/ijms.119669

**Published:** 2026-10-02

**Authors:** Yu-Wei Chiang, Hsuan-Ying Chen, Yi-Fu Huang, Cheng-Pu Hsieh

**Affiliations:** 1 Department of Orthopedic Surgery, Changhua Christian Hospital, Changhua, 50006, Taiwan.; 2 Orthopedics & Sports Medicine Laboratory, Changhua Christian Hospital, Changhua, 50006, Taiwan.; 3 Department of Post-Baccalaureate Medicine, College of Medicine, National Chung Hsing University, Taichung, 40227, Taiwan.; 4 Department of Kinesiology, Health and Leisure Studies, Chien Kuo Technology University, Changhua, 50094, Taiwan.

**Keywords:** fibrosis, muscle function recovery, muscle contusion, muscle regeneration, rosmarinic acid

## Abstract

Muscle contusion is the common injuries and present a challenge in traumatology. Nonsteroidal anti-inflammatory drugs (NSAIDs) relieve inflammation and pain after muscle injury, but the adverse effects including gastrointestinal bleeding and allergy are thorny problems. Therefore, it is imperative to develop safe and effective treatments. Rosmarinic acid (RA), an ester of caffeic acid, is first isolated from rosemary plant. It exhibits various biological activities, but its therapeutic potential on injured muscle remains unclear. In the study, we aimed to explore the effects of rosmarinic acid on skeletal muscle contusion. C57BL/6 mice were randomly assigned to control, injury, injury + diclofenac, and injury + RA groups. The injury of right gastrocnemius muscle was developed using a drop-mass method, and mice received phosphate-buffered saline (PBS), diclofenac (10 mg/kg BW), or rosmarinic acid (40 mg/kg BW) via intraperitoneal administration for 7 or 14 days after injury. Data revealed that rosmarinic acid treatment significantly decreased inflammatory response and collagen deposition in the injured muscles. And, rosmarinic acid treatment attenuated the levels of muscle damage markers (CK, LDH, and GOT) in serum after injury. In the injury + RA group, the levels of desmin, one of known myogenic marker, and the number of centrally nucleated myofibers, which are indicative of regenerating fibers, were both increased in injured muscle on day 7 after injury. Furthermore, rosmarinic acid treatment promoted recovery of locomotor abilities in the behavior analysis. Based on these findings, we suggested that rosmarinic acid treatment mitigated inflammatory responses to protect muscles from further damage, decreased fibrosis, enhanced muscle regeneration, and ultimately improved motor function recovery. Thus, RA may be potential drug for treating contusion-induced muscle injuries.

## Introduction

Muscle contusion is the common injuries and present a challenge in traumatology. The healing process of injured muscle consists of several phases, including degeneration, inflammation, regeneration, and remodeling. Inflammation plays a critical role in this process. Following muscle injury, inflammatory response is initiated through the recruitment and activation of inflammatory cell 1, 2. A well-regulated inflammatory response promotes satellite cells activation and supports muscle regeneration 3, 4. However, excessive or prolonged inflammatory responses may stimulate fibrotic remodeling, leading to excessive collagen deposition and fibrosis 5, which subsequently hinder muscle regeneration and impairs functional recovery 6, 7.

Current conventional treatments for skeletal muscle injuries include the RICE protocol (rest, ice, compression, and elevation) , immobilization, corticosteroids, and non-steroidal anti-inflammatory drugs (NSAIDs)^8^. NSAIDs are the cyclooxygenase-1 and cyclooxygenase-2 (COX-2) blocker, and commonly used to relieve inflammation and short-term pain 9. However, the adverse effects such as peptic ulcers, gastrointestinal bleeding, allergy, and deteriorated renal function are thorny problems 10-12. Therefore, it is imperative to develop safe and effective treatments.

Rosmarinic acid is an ester of caffeic acid that first isolated from the rosemary plant (*Rosmarinus officinalis L.*) in 1958 13. It has demonstrated to have various biological activities. For example, romairnic acid decreases the levels of lipid peroxidation and nitric oxide induced by lipopolysaccharide 14. Additionally, romairnic acid mitigates selenite-induced cataract damage by elevating the levels of anti-oxidant enzyme (SOD, HO1, GSH, and catalase) in rats 15. Furthermore, it exhibits anti-inflammatory activities by inhibiting formation of COX-2, thereby reducing arachidonic acid synthesis, and subsequently lowering prostaglandin E2 (PGE2) levels 16-18. Rosmarinic acid also possesses anti-tumor activities by inhibiting proteasome pathway, cell proliferations, and invasion in pancreatic and hepatocellular carcinoma 19, 20. However, the therapeutic potential of rosmarinic acid on injured muscle remains unclear. In the study, we aim to explore the therapeutic potential of rosmarinic acid on muscle contusion.

## Materials and Methods

### Animals

All C57BL/6 mice that weighted 25-30 g were male (8 weeks old), and purchased from the National Laboratory Animal Center (Taipei, Taiwan). The mice were allowed one week to acclimate to environment before testing, and were randomly divided into four groups: (1) control group (Mice were treated with phosphate-buffered saline (PBS) by intraperitoneal injection without injury), (2) injury group (Mice were treated with PBS by intraperitoneal injection after drop-mass-induced muscle injury) (3) injury + diclofenac group (Mice were treated with diclofenac (10 mg/kg body weight) by intraperitoneal injection after drop-mass-induced muscle injury), and (4) injury + RA group (Mice were treated with rosmarinic acid (RA) (40 mg/kg body weight) by intraperitoneal injection after drop-mass-induced muscle injury).

The dose of rosmarinic acid (40 mg/kg body weight) was chosen based on previous reports 21-23. Diclofenac (purity≧ 99%) was obtained from Cayman Chemical (Ann Arbor, Michigan, USA), and rosmarinic acid (purity≧ 98%) was obtained from ChemFaces (Wuhan, China). All animal experiments conducted with humane care in accordance with Animal Research: Reporting of *In vivo* Experiments (ARRIVE) guidelines and the Institutional Animal Care and Use Committee (IACUC) guidelines of Changhua Christian Hospital (CCH-AE-113-004).

The sample size (n= 6 per group) was determined using the resource equation approach 24, 25 in compliance with ARRIVE guidelines and 3R ethics principles. For a 4-group ANOVA design, n= 6 yields an error degree of freedom (DF) of 20, which falls within the optimal range (10< DF< 20) to guarantee statistical reliability while minimizing animal usage.

### Induction of drop-mass-induced muscle injury

Mice were placed prone position with isoflurane anesthetized, and the hair on the right leg was shaved using a hair clipper. A 50 g weight was free-fallen from an elevation of 40 cm through a plastic tube onto the right gastrocnemius muscle. Then, mice were monitored until it has recovered from anesthesia. The injury procedure was according our previous study 26.

### Open field test

The open field test was conducted in a dark, quiet room before injury (day 0), and on day 3, 7 and 14 after injury. Each mouse was placed in the center of a behavior box (40 × 40 × 30 cm, length × width × height), and spontaneous activity was recorded for 5 minutes. The movement trajectories were recorded using the Ethovision XT video tracking system (Noldus Information Technology, the Netherlands) to measure the total moving distance.

### Stride length analysis

The hind feet of the mice were coated with blue ink to capture their footprints as they walked across a paper-lined tracker in a dimly lit environment (60 × 60 × 300 mm, width × height × length). The distance between the 2nd to 4th footprints on the injured side was measured as the stride length.

### Accelerating rota-rod test

The latency to fall was recorded by a rota-rod device (YLS-31A, Yiyan S&T Development Co., Ltd., Jinan, Shandong, China). Testing was conducted before injury (day 0), day 3, 7, and 14 after injury. The rod speed was set gradually accelerated from 4 rpm to 30 rpm over 5 minutes within a trial. A longer latency indicates better motor ability.

### Blood biochemical assessments

On day 7 and 14 after injury, mice were sacrificed via CO₂ inhalation, and blood was immediately collected via cardiac puncture. Serum glutamate pyruvate transaminase (GPT), glutamate oxaloacetate transaminase (GOT), creatine kinase (CK), and lactic dehydrogenase (LDH) were measured using an automatic analyzer (FUJI DRI-CHEM NX600, Japan).

### Hematoxylin and eosin (H&E) staining and analysis

The 4% paraformaldehyde-fixed and paraffin-embedded tissues were cut into 5 μm sections, and stained with hematoxylin and eosin (H&E). Myofiber regeneration was quantitatively assessed on H&E-stained sections. For each animal, three independent fields per animal were captured using an optical microscope (Olympus BX 61, Olympus, Japan). The total number of myofibers in each field was counted. Regenerating myofibers were identified by the presence of centrally located nuclei, whereas mature myofibers were identified by peripherally located nuclei. The numbers were manually counted using Cell Counter plugin in ImageJ/FIJI software (National Institutes of Health, Bethesda, MD, USA).

### Immunohistochemistry staining and analysis

The 4% paraformaldehyde-fixed and paraffin-embedded tissue were cut into 5 μm sections, and immunohistochemical staining was performed for CD11b (Ab133357, 1:100, Abcam, USA), NF-κB p65 (#8242, 1:100, Cell Signaling Technology, USA), TGF-β (Ab215715, 1:100, Abcam, USA), p-Smad2/3 (Ab52903, 1:100, Abcam, USA), desmin (Ab15200, 1:100, Abcam, USA), and Pax7 (AB528428, 1:100, DSHB, USA). Positive signals were detected using DAB staining, followed by counterstaining with hematoxylin for nuclear. Images of CD11b, NF-κB p65, TGF-β, and desmin staining were captured using an optical microscope, and the positive signals were subsequently quantified using the Color Deconvolution (H-DAB) plugin in ImageJ/FIJI software with a fixed threshold of 200. Images of p-Smad2/3 and Pax7 staining were captured using an optical microscope, and the number of positive cells was counted using the Cell Counter plugin in ImageJ/FIJI software.

### Masson's trichrome staining and analysis

Masson's trichrome staining was performed to evaluate collagen deposition, which can reflect the degree of fibrosis 27. Tissues were cut into 5 μm sections, and were stained with a Masson's Trichrome Kit (ScyTek Laboratories, Logan, Utah, USA) according to the manufacturer's instructions. After deparaffinization and rehydration, tissue sections were mordanted in Bouin's solution at 56°C for one hour. The sections were subsequently immersed in Biebrich scarlet-acid fuchsin for cytoplasmic staining, followed by differentiation in phosphomolybdic-phosphotungstic acid. Collagen fibers were then specifically highlighted by staining with aniline blue before a final stabilization step in 1% acetic acid. The stained sections revealed blue collagen fiber, and red muscle fiber for the evaluation of the area of collagen. Image was captured using an optical microscope. All images were analyzed using ImageJ/FIJI software. The Color Deconvolution (Masson trichrome) plugin was utilized with a fixed threshold of 180, and results were expressed as the ratio of collagen area to total tissue area.

### Statistical analysis

Data in present research are presented as means ± standard error of the mean (SEM). Statistical analysis was conducted using one-way ANOVA followed by Duncan's post hoc test. Behavioral data (distance, stride length, and latency time) were analyzed using a mixed-effects model (REML) with treatment and time as fixed effects and subject as a random effect, followed by Tukey's multiple-comparisons test. A *p*-value of < 0.05 was considered to indicate a statistically significant difference.

## Results

### Rosmarinic acid decreases the levels of muscle damage markers after muscle contusion

Muscle contusion in mice was established by drop-mass method. 24 mice were randomly assigned to four groups (6 mice/group), namely: (1) control group, mice were treated with phosphate-buffered saline (PBS) without injury; (2) injury group, mice were treated with PBS after drop-mass-induced muscle injury; (3) injury + diclofenac group, mice were treated with diclofenac (10 mg/kg body weight) after drop-mass-induced muscle injury; (4) injury + RA group, mice were treated with rosmarinic acid (RA) (40 mg/kg body weight) after drop-mass-induced muscle injury (Figure 1). The changes of body weight were recorded during the experimental period, and there were no significant differences among groups after drugs treatment (Figure 2A). Serum glutamate pyruvate transaminase (GPT) is usually used to monitor drug-induced liver injury 28. GPT levels were approximately 42-48 U/L, and there were no differences among groups after drug treatment (Figure 2B). Thus, rosmarinic acid should be safe for further application.

The levels of creatine kinase (CK), lactate dehydrogenase (LDH), and glutamic oxaloacetic transaminase (GOT) in serum are used as damage markers after muscle injury. As shown in Figure 3A, the levels of CK in serum were approximately 210 U/L in the control group. And, the levels were increased to 690 U/L on day 7 and changed to approximately 410 U/L on day 14 after injury. In the injury + diclofenac group, CK levels were increased to about 360 U/L on day 7 and changed to approximately 285 U/L on day 14 after injury. In contrast, in the injury + RA group, CK levels were about 194 U/L on day 7 and changed to approximately 250 U/L on day 14 after injury. And there were no significant differences between the control group and the injury + RA group either on day 7 or day 14 after injury. Similar to CK levels, LDH and GOT levels were increased by muscle injury, and these elevated levels were attenuated in the injury + diclofenac and injury + RA group (Figure 3B and 3C). Those findings indicate that rosmarinic acid treatment could effectively alleviate early increase of damage markers after muscle injury.

### Rosmarinic acid promotes recovery of locomotor activity and motor ability after muscle contusion

Limb damage and pain cause a decline in behavioral performance. This decline can be alleviated when the damage or pain is reduced 26, 29. Open field test and stride length analysis are used to assess locomotor activity. In open field test, the total moving distance (% of day 0) was significantly decreased after injury, but the decreases were recovered in the injury + diclofenac and injury + RA groups (Figure 4A). Stride length is the distance between the second to fourth footprints. The stride length was shorter in the injury group after muscle injury, and the shortened distance was rescued in the injury + diclofenac and injury + RA groups (Figure 4B).

Rota-rod test is applicable to study the change of motor ability. In this test, latency time is the time that mice remain on a rotating rod before falling off. A longer latency time is associated with better motor ability. The latency time were significantly decreased after injury in the injury group, and the decreased time was recovered in the injury + diclofenac and injury + RA groups (Figure 5). These data suggest that rosmarinic acid effectively restores locomotor activity and motor ability after muscle contusion.

### Rosmarinic acid mitigates inflammatory response and collagen deposition, and enhances muscle regeneration after muscle contusion

Inflammation is the early response after muscle injury, characterized by neutrophil infiltration and subsequent macrophage activation to clear damaged tissues. Excessive or persistent inflammation during muscle injury contributes to fibrosis by promoting collagen deposition, and thus impairs muscle regeneration 30. To demonstrate whether rosmarinic acid affected inflammatory response, the levels of CD11b, a marker for myeloid-derived inflammatory cells including neutrophils, monocytes, and macrophages 31, 32, were analyzed in the injured muscles. The results showed that treatment with rosmarinic acid decreased the levels of CD11b in the injured muscles (Figure 6A and 6B) that accompanied by the downregulation of NF-κB p65 expression (Figure 6A and 6C). These results indicate that rosmarinic acid could suppress inflammatory response in the injured muscles.

Furthermore, Masson's trichrome staining was used to evaluate collagen deposition (blue color) in injured muscles on day 14 after injury. As showed in Figure 6D and 6E, the collagen area from injured muscles was less in injury + RA group compared to the injury and injury + diclofenac groups. In addition, the TGF-β/Smad pathway which plays a central mediator of fibrogenesis was downregulated in injured muscles in injury + RA group compared to the injury group (Figure 6F, 6G, and 6H). These data suggest that rosmarinic acid could have ability to suppress collagen deposition.

Muscle regeneration typically occurs during the first 7-10 days after injury. To evaluate muscle regeneration, immunohistochemical staining for desmin, one of known myogenic marker, was performed on day 7 after muscle injury. The data displayed that the level of desmin was elevated after injury, and its level in the injury+ RA group was higher compared to the levels in the injury and injury+ diclofenac groups (Figure 7A and 7B).

During early stage of muscle regeneration, satellite cells were activated to form new myofibers. These regenerating myofibers have nuclei that are positioned in the center of the fiber. And, during the later stage of regeneration, regenerating myofibers mature to form normal sized myofibers by fusion. These mature myofibers are characterized by multiple nuclei that are peripheral to the fiber 33. As shown in Figure 7, on day 7 after injury, Pax7 expression (Figure 7A and 7C), a marker of muscle satellite cells, and the total number of myofibers (Figure 7D and 7E) and centrally nucleated myofibers (Figure 7D and 7F) were significantly increased in the injury + RA group compared to the injury and injury + diclofenac groups.

Furthermore, on day 14 after injury, total number of myofibers were decreased, but the number of peripherally nucleated myofibers were increased in the injury + RA group compared to the number in the injury and injury+ diclofenac groups (Figure 8A, 8B and 8C). These findings suggest that rosmarinic acid may have an effective ability to improve muscle regeneration after muscle contusion.

## Discussion

Muscle contusion is common injury resulting from blunt impact. The main treatment is RICE protocol (rest, ice, compression, and elevation) during acute phase to minimize swelling, bleeding, and further damages. Non-steroidal anti-inflammatory drugs (NSAIDs) are prescribed to decrease inflammation and pain, but the harassed side effects such as gastrointestinal symptoms and allergy need to overcome. The present study exhibited that rosmarinic acid treatment alleviated inflammatory response and collagen deposition, deceased the levels of muscle damage markers (CK, LDH, and GOT), enhanced muscle regeneration, and further improved muscle functional recovery. Creatine kinase (CK), lactate dehydrogenase (LDH), and glutamic oxaloacetic transaminase (GOT) are intracellular muscular enzymes commonly used as biomarkers to assess muscle injury 34-36. Previous study indicates that CK and GOT levels are elevated after muscle damage, and the lower level has a positive effect on the early recovery 37.

Pro-inflammatory cytokines recruit neutrophils and neutrophil accumulation in injured muscle is considered to be the evidence of an inflammatory response after contusion injury 1, 38. PGE2 is associated with inflammation and pain. Rosmarinic acid may exhibit excellent anti-inflammatory activity and analgesic potential 39, 40 by lowering PGE2 levels 16-18. We analyzed behavior changes of walking distance and stride length which are related to pain and muscle recovery after injury. Data showed that the reduction in walking distance and stride length induced by injury were recovered following rosmarinic acid treatment. Additionally, the pathological data revealed that inflammatory response were lower in rosmarinic acid-treated groups (see Figure 6A). Thus, we suggested that romarinic acid may alleviate pain and inflammatory response, reduce the levels of muscle damage biomarkers, and protect muscle from further injury. These findings were consistent with the anti-inflammatory and analgesic properties of rosmarinic acid.

Inflammation triggers formation of collagen fiber to support healing, but excessive collagen fibers lead to fibrotic scar tissue, which impairs muscle regeneration and functional recovery 7, 41. The anti-fibrosis activity of rosmarinic acid in lungs, liver, kidneys, and heart has been explored 42-45. Our data showed that rosmarinic acid treatment reduced fibrotic area by 50 % compared to the injury group in the injured muscle that induced by contusion (on day 14 after injury, see Figure 6D). Furthermore, rosmarinic acid treatment resulted in the downregulation of TGF-β/Smad pathway, a central mediator of fibrogenesis, in the injured muscle (Figure 6F). The present study was the first to exhibit anti-fibrosis effect of rosmarinic acid on muscle contusion. Fibrosis can be regulated by various factors. How rosmarinic acid influence fibrosis in injured muscle requires further clarification.

Desmin is a myogenic marker during myogenesis in the early stage after muscle injury 6. The higher levels of desmin are effective in muscle repair 46, 47. Our data showed that rosmarinic acid treatment increased the levels of desmin after muscle injury. In addition, we also found Pax7 expression, a marker of muscle satellite cells, and the numbers of total myofibers and centrally nucleated myofibers (as regenerated myofibers) were significantly higher in rosmarinic acid-treated group on day 7 after injury (Figure 7). And, more satellite cell rings were observed in injured muscle in the romarinic acid-treated group (Figure 7D, white arrow). Satellite cell rings, the concentric arrangements of satellite cells around muscle fibers, were considered to be the signs of early regeneration and could be correlated with higher regenerative capacity 48. According to these results, we suggested that rosmarinic acid may increase myogenesis, and then enhance muscle regeneration.

In mouse muscle injury models, the acute inflammatory response, satellite cell activation, and myofiber regeneration typically peak within the first week, followed by the onset of functional recovery from days 7 and 14 49, 50. Therefore, the 14-day time point used in this study appropriately captures the acute-to-early regenerative phase of muscle repair. Although the efficacy of rosmarinic acid has been confirmed within 14-day observation period in our study, longer observation periods (e.g., 28 days) in future studies will be advantageous to evaluate its long-term consequences on chronic fibrosis and permanent functional recovery.

Rosmarinic acid has different properties such as anti-inflammation, anti-oxidation, and anti-fibrotic activity 16-18, 42-45**,** but only few studies have explored the properties on muscle anabolism 51-54. Shang et al. indicate that dietary supplementation of rosmarinic acid raises the levels of total protein in serum and improves the growth performance in white-feathered broilers 51. Rosmarinic acid supplementation enhances muscle mass and exercise performance by promoting myogenic differentiation, and muscle synthesis in mice and C2C12 cell line models 52. Chen et al. find that rosmarinic acid protects C2C12 myoblast differentiation from heat damage, and maintains formation of myotube 53. Furthermore, rosmarinic acid may against muscle weakness and atrophy by activating the AKT/p70S6K/mTOR signal pathway and reducing the production of reactive oxygen species and inhibiting apoptosis in dexamethasone-treated C2C12 myotubes 54. In this study, our data provided evidences to support effective effects of rosmarinic acid on muscle regeneration after muscle contusion. This is important for the development of drugs targeting muscle-related diseases.

Finally, there were several limitations in this study. First, CD11b was used as a marker for myeloid-derived inflammatory cells. It cannot distinguish different types of immune cells. Future studies incorporating lineage-specific and polarization markers would further clarify the immune cell dynamics during muscle repair. Second, the assays to directly evaluate muscle strength were missing. Although the rotarod test and locomotor analyses provide evidence of improved motor performance, these assessments primarily reflect integrated motor coordination rather than direct muscle strength. Third, more evidences or further analyses to support the verified signaling pathways were missing.

## Conclusion

Our data showed that rosmarinic acid treatment attenuated inflammatory response to protect muscle from further damage, decreased the fibrotic area, enhanced muscle regeneration, and ultimately improved motor function recovery after muscle contusion. Rosmarinic acid may be potential drug for treating contusion-induced muscle injuries.

## Figures and Tables

**Figure 1:**
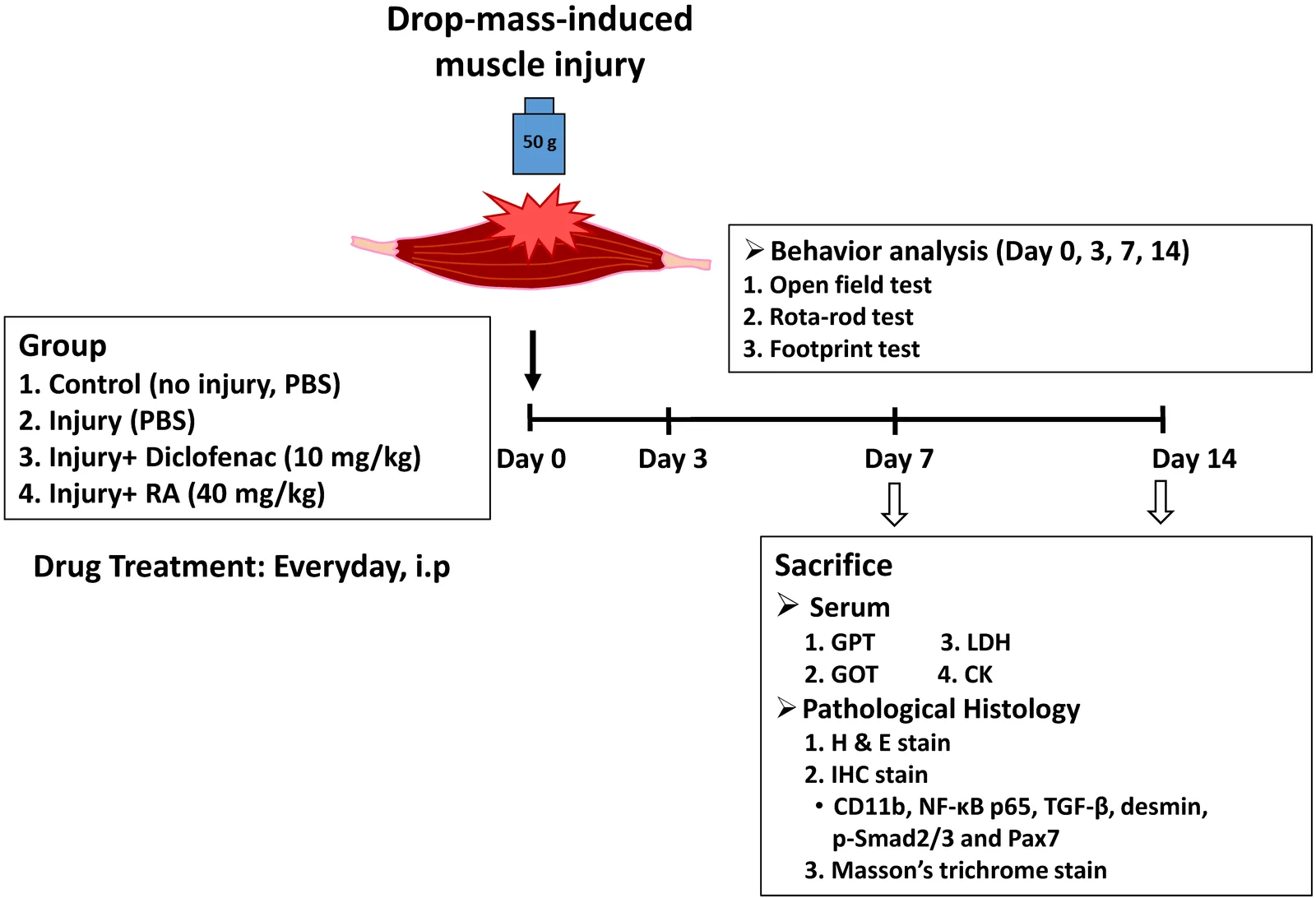
The flow chart of the study design after drop-mass-induced muscle injury in mice.

**Figure 2:**
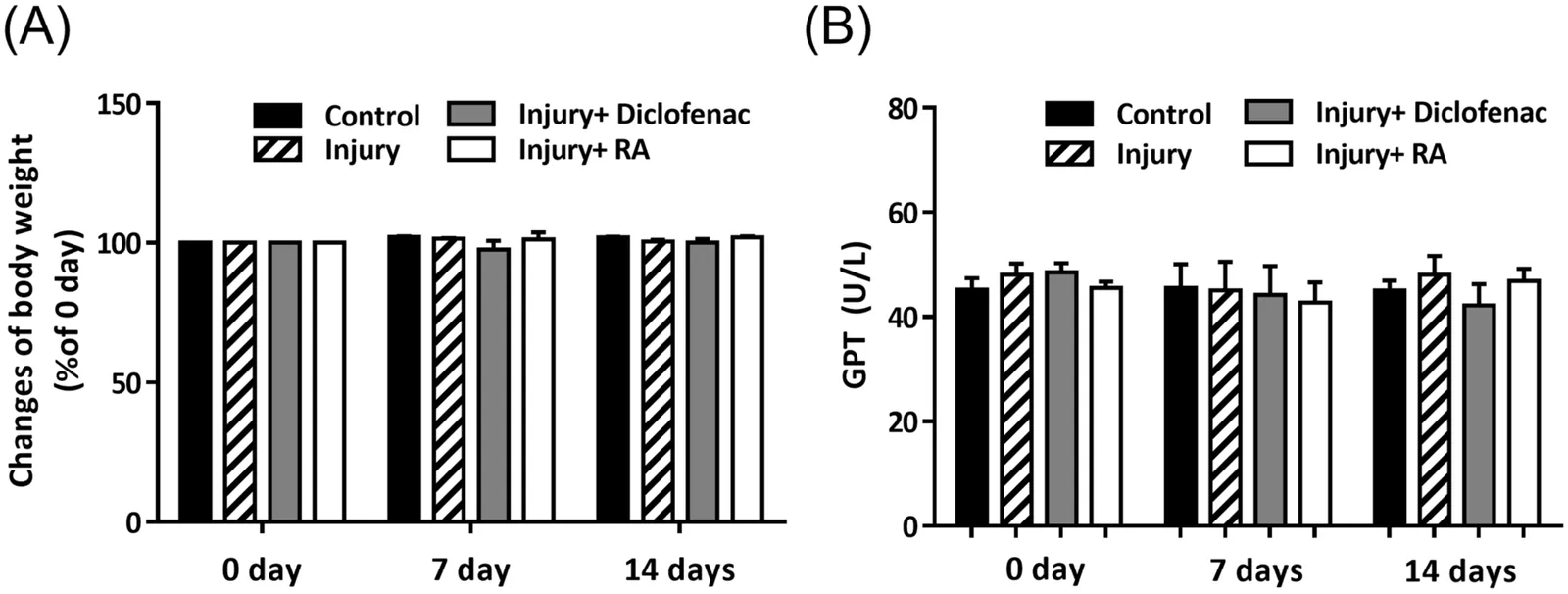
** The safety assessment of rosmarinic acid (RA) treatment.** 24 mice were randomly assigned to four groups, namely: (1) control group (Mice were treated with PBS by intraperitoneal injection without injury), (2) injury group (Mice were treated with PBS by intraperitoneal injection after drop-mass-induced muscle injury) (3) injury + diclofenac group (Mice were treated with diclofenac (10 mg/kg body weight) by intraperitoneal injection after drop-mass-induced muscle injury), and (4) injury + RA group (Mice were treated with rosmarinic acid (RA) (40 mg/kg body weight) by intraperitoneal injection after drop-mass-induced muscle. **(A)** The changes of body weight. Body weights were recorded and presented as % of 0 day. **(B)** Serum GPT levels. Serum GPT levels were analyzed on day 0, 7 and 14 after injury. Data were presented as the mean ± SEM.

**Figure 3:**
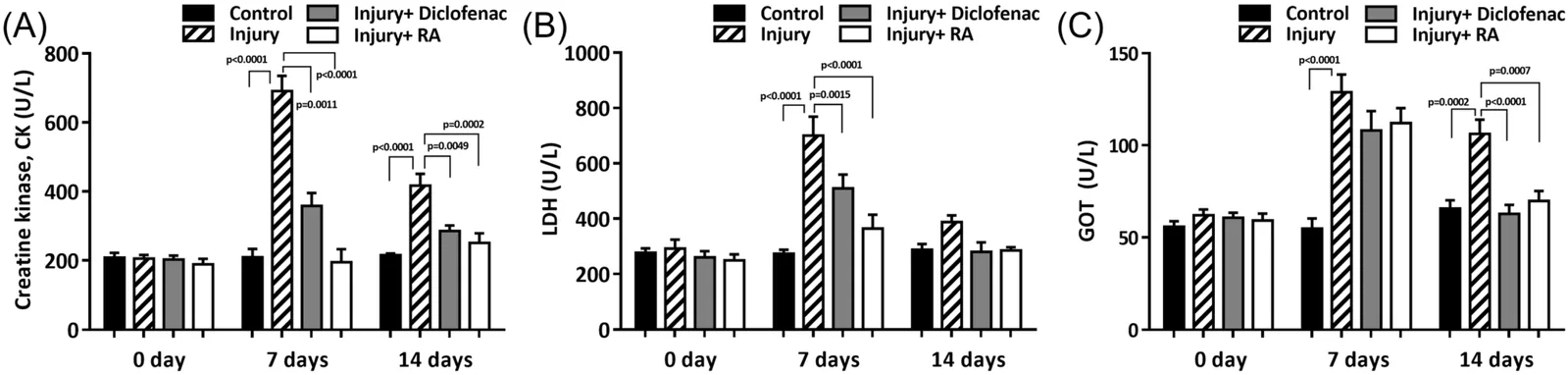
** Rosmarinic acid (RA) decreases muscle injury markers after muscle contusion. 24** mice were randomly assigned to four groups, namely: (1) control group, (2) injury group, (3) injury + diclofenac group, and (4) injury + RA group. The details of each group were described in Figure 2. Serum CK **(A)**, LDH **(B)**, and GOT **(C)** were analyzed on day 0, 7 and 14 after injury. Data were presented as the mean ± SEM.

**Figure 4:**
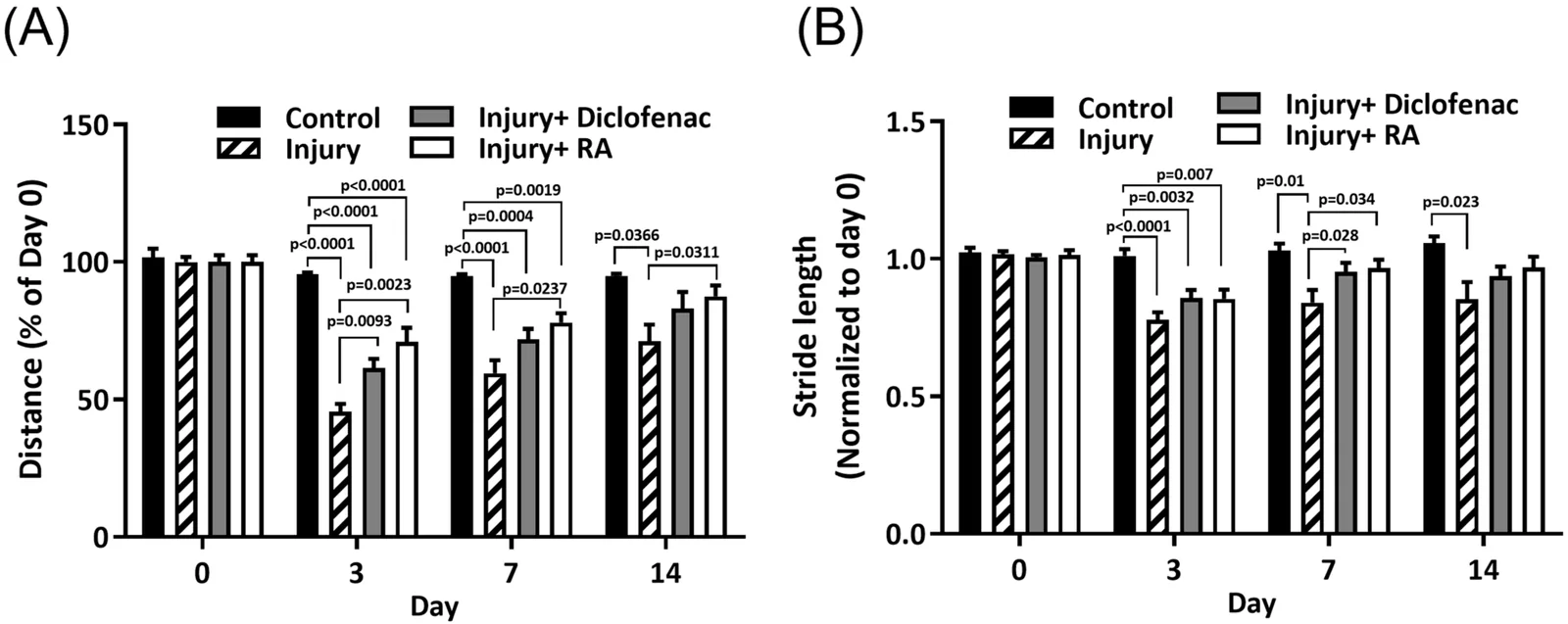
** Rosmarinic acid (RA) restores locomotor activity after muscle contusion. 24** mice were randomly assigned to four groups, namely: (1) control group, (2) injury group, (3) injury + diclofenac group, and (4) injury + RA group. The details of each group were described in Figure 2. Locomotor activities were assessed by open field test **(A)** and stride length analysis **(B)** on day 0, 3, 7, and 14 after injury. Data were presented as the mean ± SEM.

**Figure 5:**
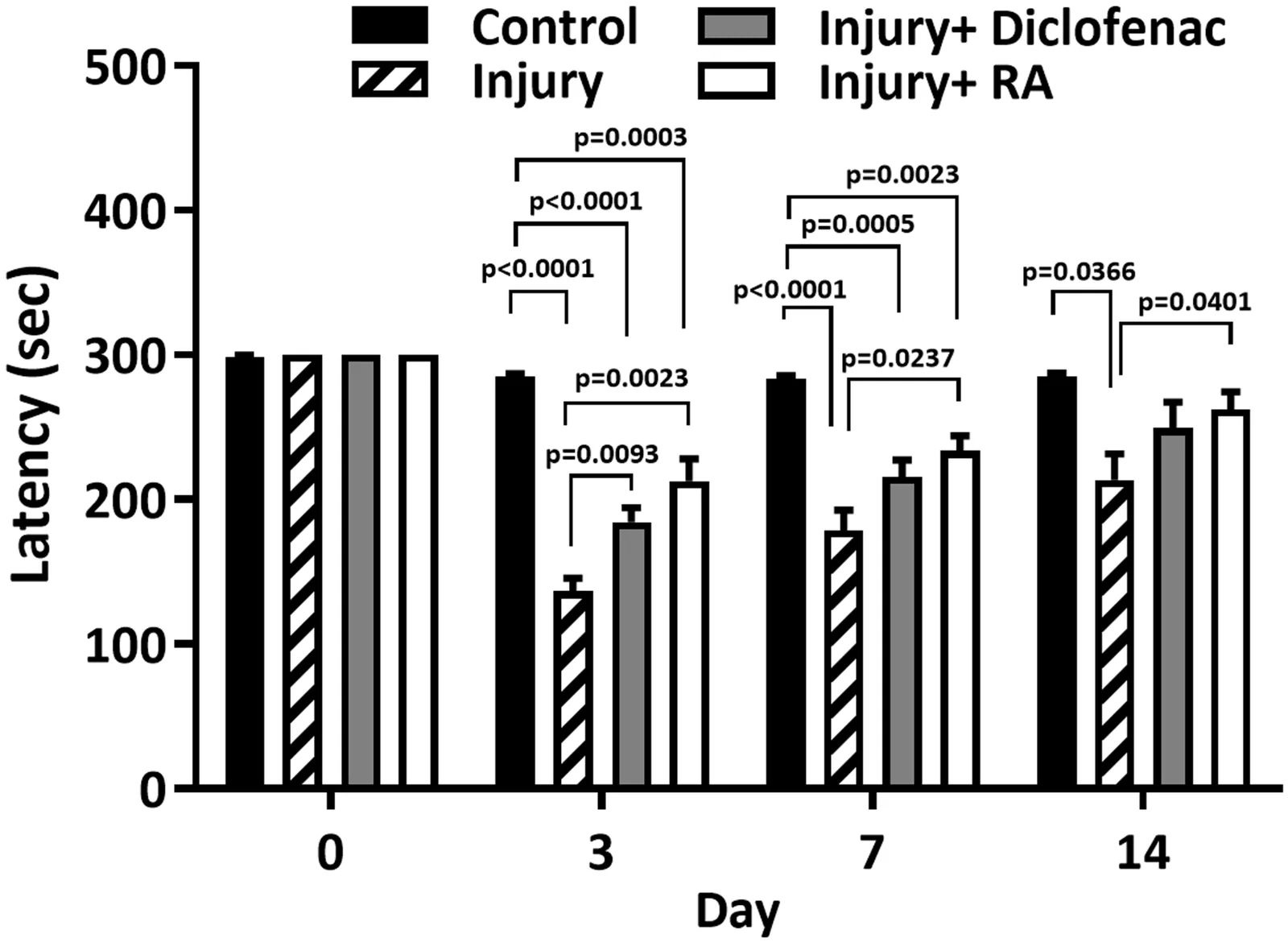
** Rosmarinic acid (RA) recovers motor ability after muscle contusion.** 24 mice were randomly assigned to four groups, namely: (1) control group, (2) injury group, (3) injury + diclofenac group, and (4) injury + RA group. The details of each group were described in Figure 2. Rota-rod test were performed on day 0, 3, 7, and 14 after injury to assess the motor ability. Data were presented as the mean ± SEM.

**Figure 6:**
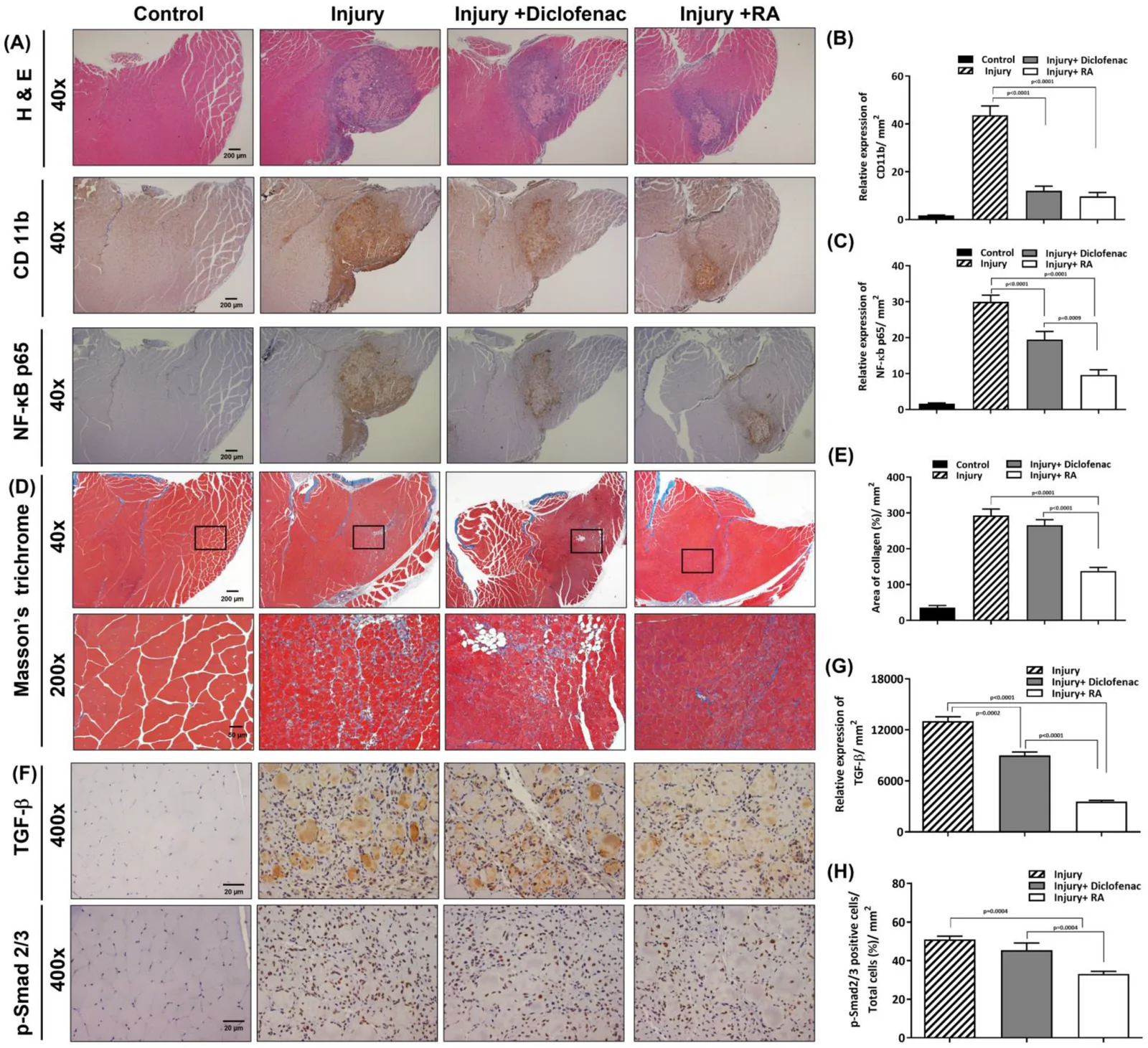
** Rosmarinic acid (RA) attenuates the infiltration of inflammatory cells and collagen deposition after muscle contusion.** 24 mice were randomly assigned to four groups, namely: (1) control group, (2) injury group, (3) injury + diclofenac group, and (4) injury + RA group. The details of each group were described in Figure 2. **(A)** For pathological histology analysis, gastrocnemius muscle samples were stained by hematoxylin and eosin (H&E) on day 7 after injury. Immunohistochemistry analysis was performed to investigate the expression of CD11b **(B)** and NF-κB p65 **(C)**. The results are expressed as mean ± SEM from six microscopic fields/group (magnification 40×) derived from six animals (one field per single animal). **(D and E)** Collagen areas on sections of muscle samples were analyzed by Masson's trichrome staining on day 14 after injury. Collagen fibers revealed blue and muscle fibers revealed red. Selected zone on the image was enlarged and presented (magnification 200×). Area of collagen was quantified. The results are expressed as mean ± SEM from six microscopic fields/group (magnification 40×) derived from six animals (one field per single animal). **(F)** TGF-β and p-Smad2/3 expression on sections of muscle samples was determined by immunohistochemistry analysis on day 14 after injury. The results are expressed as mean ± SEM from 36 microscopic fields/group (magnification 400×) derived from six animals (six independent fields per single animal) **(G and H)**.

**Figure 7:**
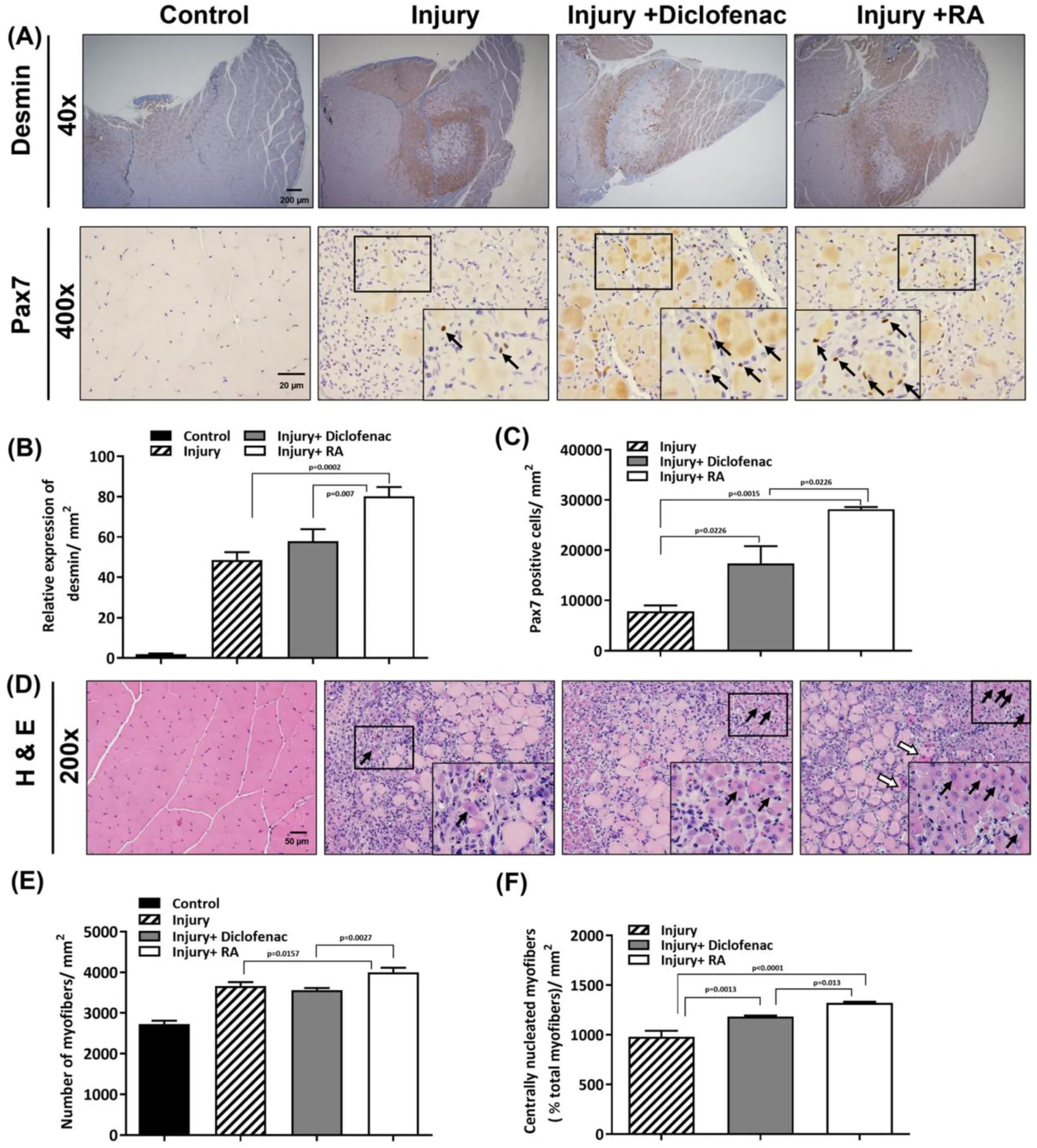
** Rosmarinic acid (RA) enhances desmin expression and the number of regenerating myofibers with central nucleus on day 7 after muscle contusion.** 24 mice were randomly assigned to four groups, namely: (1) control group, (2) injury group, (3) injury + diclofenac group, and (4) injury + RA group. The details of each group were described in Figure 2. Gastrocnemius muscle samples were collected and analyzed on day 7 after injury. **(A)** Immunohistochemistry analysis showed the expression of desmin and Pax7. Selected zone on the image was enlarged and presented. Relative expression of desmin was quantified. The results are expressed as mean ± SEM from six microscopic fields/group (magnification 40×) derived from six animals (one field per single animal) **(B)**. Number of Pax7-positive cells (black arrow) were counted. The results are expressed as mean ± SEM from 36 microscopic fields/group (magnification 400×) derived from six animals (six independent fields per single animal) **(C)**. **(D)** H&E staining was performed to observed total number of myofibers **(E)** and the number of centrally nucleated myofibers **(F)**. Selected zone on the image was enlarged and presented. Black arrow indicates centrally nucleated myofibers. White arrow indicates satellite cell rings. Number of myofibers were counted. The results are presented as mean ± SEM from 18 microscopic fields/group (magnification 200×) derived from six animals (three independent fields per single animal).

**Figure 8:**
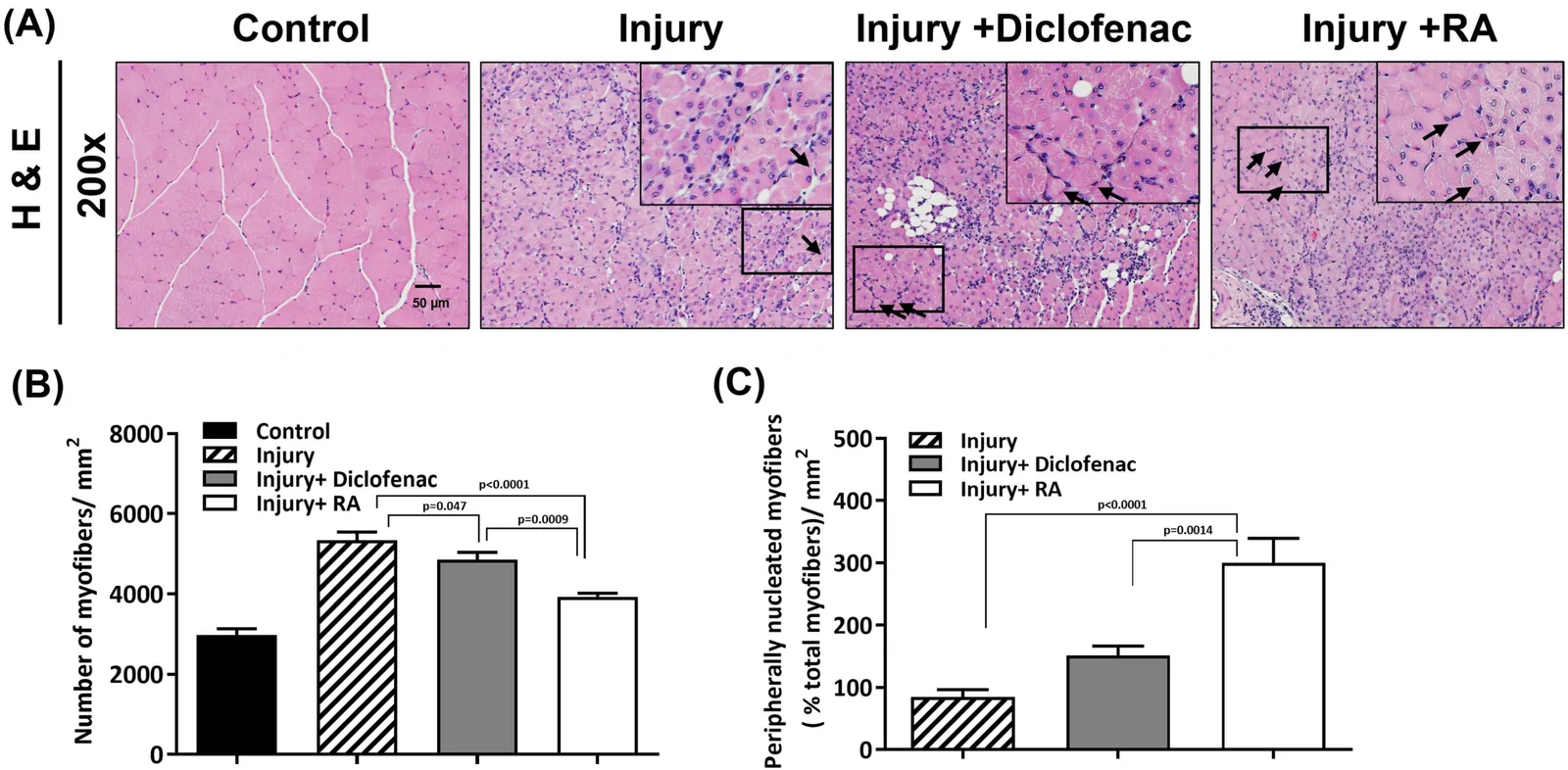
** Rosmarinic acid (RA) increases the number of mature myofibers with multiple nuclei at the periphery of the fibers on day 14 after muscle contusion.** 24 mice were randomly assigned to four groups, namely: (1) control group, (2) injury group, (3) injury + diclofenac group, and (4) injury + RA group. The details of each group were described in Figure 2. Gastrocnemius muscle samples were collected and analyzed on day 14 after injury. **(A)** H&E staining were performed to observe total number of myofibers **(B)** and the number of peripherally nucleated myofibers **(C)**. Selected zone on the image was enlarged and presented. Black arrow indicates mature myofiber with peripheral nuclei. Number of myofibers were counted. The results are presented as mean ± SEM from 18 microscopic fields/group (magnification 200×) derived from six animals (three independent fields per single animal).

## Data Availability

Data of current study were available from the corresponding author on reasonable request.
